# Unsupervised Action Proposals Using Support Vector Classifiers for Online Video Processing

**DOI:** 10.3390/s20102953

**Published:** 2020-05-22

**Authors:** Marcos Baptista Ríos, Roberto Javier López-Sastre, Francisco Javier Acevedo-Rodríguez, Pilar Martín-Martín, Saturnino Maldonado-Bascón

**Affiliations:** GRAM, Department of Signal Theory and Communications, University of Alcalá, 28805 Alcalá de Henares, Spain; robertoj.lopez@uah.es (R.J.L.-S.); javier.acevedo@uah.es (F.J.A.-R.); p.martin@uah.es (P.M.-M.); saturnino.maldonado@uah.es (S.M.-B.)

**Keywords:** action proposals, action recognition, computer vision, unsupervised learning, intelligent video sensor

## Abstract

In this work, we introduce an intelligent video sensor for the problem of Action Proposals (AP). AP consists of localizing temporal segments in untrimmed videos that are likely to contain actions. Solving this problem can accelerate several video action understanding tasks, such as detection, retrieval, or indexing. All previous AP approaches are supervised and offline, i.e., they need both the temporal annotations of the datasets during training and access to the whole video to effectively cast the proposals. We propose here a new approach which, unlike the rest of the state-of-the-art models, is unsupervised. This implies that we do not allow it to see any labeled data during learning nor to work with any pre-trained feature on the used dataset. Moreover, our approach also operates in an online manner, which can be beneficial for many real-world applications where the video has to be processed as soon as it arrives at the sensor, e.g., robotics or video monitoring. The core of our method is based on a Support Vector Classifier (SVC) module which produces candidate segments for AP by distinguishing between sets of contiguous video frames. We further propose a mechanism to refine and filter those candidate segments. This filter optimizes a learning-to-rank formulation over the dynamics of the segments. An extensive experimental evaluation is conducted on Thumos’14 and ActivityNet datasets, and, to the best of our knowledge, this work supposes the first unsupervised approach on these main AP benchmarks. Finally, we also provide a thorough comparison to the current state-of-the-art supervised AP approaches. We achieve 41% and 59% of the performance of the best-supervised model on ActivityNet and Thumos’14, respectively, confirming our unsupervised solution as a correct option to tackle the AP problem. The code to reproduce all our results will be publicly released upon acceptance of the paper.

## 1. Introduction

In the extensive field of video understanding, the problem of Temporal Action Detection (TAD) in untrimmed videos has gained massive interest in the recent years, as evidenced by the great amount of work that has been proposed (e.g., [1,2,3,4,5,6,7,8,9]). For a recent survey on the topic, we refer the reader to [10].

Currently, the best and most common way to solve the TAD problem involves two stages [11]: (1) Temporal Action Proposal generation (TAP); and, (2) Action Recognition. That is, the best-performing TAD solutions start with a TAP module, which is responsible for analyzing the whole video to generate temporal segments that are likely to include an action. This TAP step, which technically filters the video, is followed by an action recognition deep network, i.e., a classification deep model, that performs the action labeling on the generated segments. Among all works, different kind of classifiers have been successfully applied (e.g., SCNN [12], TSN [13] and UntrimmedNets [14]). For instance, CTAP [15] gets its best results with [12], while BSN [16], BMN [17] and MGG [18] use both [13,14]. It is also interesting to note that all winning approaches of the ActivityNet challenge follow this described two-step pipeline, as it has been reported in [11]. This fact confirms that the TAP step is crucial for the whole TAD task. It also explains the large amount of TAP works that have been published to date (e.g., [12,16,17,19,20,21,22,23,24,25]).

However, all the proposed TAP approaches address the problem from a supervised learning paradigm and with an offline setting. These conditions imply that: (1) during learning, the set of temporal annotations of action detection datasets (such as ActivityNet [26] or Thumos-14 [27]) is used; (2) the whole video must be processed before generating the action proposals. This setting leads to having lots of proposals which usually overlap and are overestimated to maximize the recall (not the precision) of the models.

**Figure 1:**
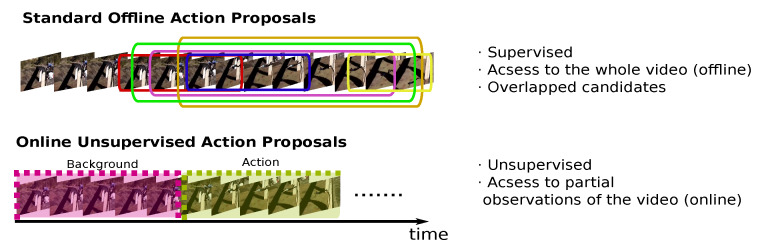
Comparison between the standard offline Action Proposals paradigm, and the novel online and unsupervised approach introduced in this work.

The work presented in this article aims to explore a new direction, see Figure 1. First, the solution is totally unsupervised. We do not allow our model to use any labeled data during training nor any feature pre-trained on the used dataset. Second, we want our solution to work online. It must be able to generate high-quality action proposals as soon as they occur in a video stream, hence working as an online video sensor. This restriction forces the model to work with partial observations of the video, instead of having access to the whole video to make and refine proposals. Such a scenario seems much more challenging, without a doubt. However, we believe that it opens the door to implementing new intelligent video sensors, capable of working online and with minimal supervision, making it easier for them to adapt to changing environments. Imagine a robot that has to interact with a human in a realistic scenario, or an intelligent video surveillance service designed to raise an alarm when an action is detected. Previous offline methods make the described applications impossible because they would detect the action situations way later than they have occurred. Moreover, if the environment changes, a model trained with a supervised setting might experience more performance problems than an unsupervised pipeline.

**Figure 2:**
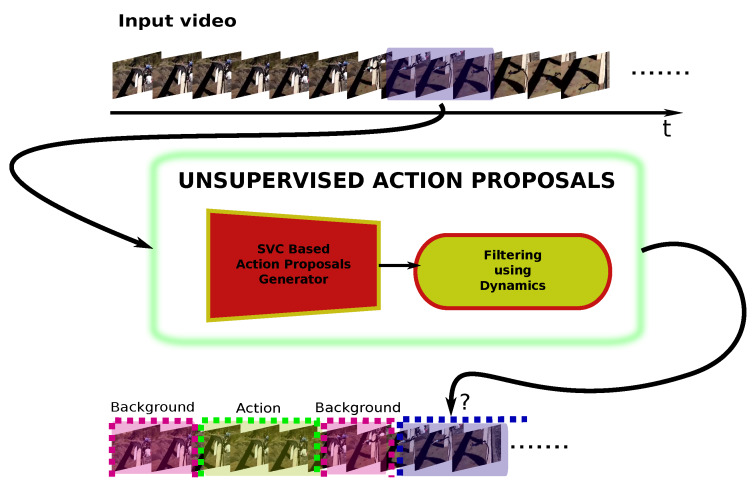
Overview of the unsupervised AP solution. It consists of an online SVC-based candidate action proposal generator, and a filtering mechanism over the dynamics of the features to consolidate the final action proposal segments. Only information until time *t* is available to the model.

Figure 2 shows an overview of the proposed approach. The main contributions of our work can be summarized as follows:

- To the best of our knowledge, we are the first in addressing the Action Proposals (AP) problem with a novel unsupervised solution, which is based on two main modules: a Support Vector Classifier (SVC) and a filter based on dynamics. While the former discriminates between contiguous sets of video frames to generate sets of candidates segments, the latter computes the dynamics of these segments and applies a distance criterion between each segment dynamics and a randomized version of them.
- Unlike all state-of-the-art approaches, ours is the first completely online. The video is processed as it arrives at the sensor, without accessing any information from the future nor modifying any past decision.
- Comparing to the state of the art our best unsupervised configuration achieves more than 41% and 59% of the performance of the best supervised model for ActivityNet and Thummos’14 datasets, respectively.

## 2. Related Work

We describe in this section the most representative contributions to the Temporal Action Proposals task, and the related topic of Online Action Detection.

### 2.1. Temporal Action Proposals

Temporal Action Proposal models aim to generate candidate temporal segments which are likely to contain an action. Nearly all proposed solutions for this task can be grouped into two styles: (a) from proposals to boundaries [12,19,20,21,22,23]; and (b) from boundaries to proposals [16,17,24,25].

First-style methods typically generate thousands of varied-length overlapped action candidate segments, which can then be confirmed or discarded as valid proposals. Once the proposal is confirmed, its boundaries (initial and ending times) are refined.

A common option to generate these candidate segments is the sliding window. In this technique, videos are densely sampled several times with temporal windows of different lengths. After sampling, each video will have been transformed to a bag of overlapped candidate segments. The authors in [12,19] initialize their TAP methods with this bag of segments. Concretely, the former learns a dictionary over the features of ground truth action segments, and at test time, all candidates generated by the sliding window are tried to be reconstructed through the learned dictionary. Those segments with low reconstruction error are confirmed as actual proposals, being the rest discarded. On the other hand, the approach in [12] relies on a multi-stage C3D [28] network. First, they propose to uniformly sample frame segments of different length. Then, they solve the TAP problem by feeding with the frame segment a C3D network that has been repurposed to discriminate only between action and background.

However, in TURN-TAP [22], the sliding window is used to first decompose the video in short contiguous, but not overlapped, windows (e.g., 16 or 32 frames) called units. Afterwards, they extract unit features and pool sets of those that are contiguous to form clips. The collected clips are considered the bag of candidate action proposals. Finally, inspired by the Faster R-CNN architecture [29], two siblings fully connected layers are trained to determine whether a certain clip belongs to an action, and to perform the regression of two offsets to refine its boundaries. This method is not the only one that has taken inspiration from the Faster R-CNN. Other works, such as those proposed by [6,23], have directly repurposed the Faster R-CNN.

Besides, there also exists [20,21]. In these works, although they use the same backbone as in [12,22] (C3D), their methods generate varied-length windows and score them simultaneously. Therefore, the video is analyzed in a single pass. After this, they refine and filter the proposals.

Regarding second-style approaches (from boundaries to proposals), they commonly find, first, the temporal boundary points by analyzing an actionness score of the video features. Then, they relate those points to each other to build the proposals.

Following this style, the recent works by Lin et al., BSN [16] and BMN [17], generate the proposals by combining detected boundaries. Concretely, BSN extract for each video three probability curves: actionness, starting and ending. Those points in the video that correspond to high ending/starting and actionness probabilities are considered boundaries. Once all boundaries are collected, they are combined to build a dense bag of overlapped candidate action proposals. The features of each candidate are given a score through a simple FC layer. After applying thresholding and non-maximum suppression, the final set of proposals is built.

Apart from all the works mentioned so far, CTAP [15] and MGG [18] leverage both styles. These methods extract, first, the unit level features from videos. Afterwards, they simultaneously build: (a) a set of candidate temporal action proposals and (b) a set of segments with high actionness frames. Therefore, each candidate segment is refined by comparing it to those of the second set with high action probability. While the former refine the boundaries by regressing offsets through a temporal convolutional network, the latter join actionness and window candidates with high temporal IoU.

It is worth noticing that all these first and second-style cited works tackle the problem from a fully supervised perspective. This means that all the data used during training are labeled. The work that we propose here goes beyond all these previous methods and seeks to solve the problem through an unsupervised approach, i.e., without using any labeled data during training nor any feature pre-trained on the used dataset.

Only two works have so far explored less supervised alternatives: Ji et al. [30] and Khatir et al. [31]. With a semi-supervised approach in [30], the authors investigate on how the performance of a model is affected when varying the amount of labels used during training. Meanwhile, the model in [31] proposes to extract proposals using an online agglomerative clustering based on distances between consecutive frame features.

In this paper, we address the problem from a different perspective, where we introduce an online video processing model based on SVCs that is able to generate action proposals in an unsupervised way. To the best of our knowledge, we present the first analysis for an unsupervised TAP method in a large-scale dataset like ActivityNet [26], and also Thumos’14 dataset [27], that works in an online fashion.

### 2.2. Online Action Detection

The problem of detecting actions in an online fashion as soon as they happen (Online Action Detection, OAD) is beginning to be explored [32,33,34,35,36,37].

The seminal work of De Geest et al.  [32] introduced Online Action Detection as a per-frame labeling task and shared different baselines. Their follow-up work [34] presented a new LSTM configuration to better learn temporal patterns in actions. A different approach, based on a reinforced encoder-decoder, is proposed by [33], where OAD is seen as action anticipation when the anticipation time is zero. The recent work of Xu et al. [35] proposed a system that benefits from future anticipation to improve the predictions on current frames. Given the fact that the OAD problem is fairly new, there is a lack of consensus regarding the conditions and metrics to be used when evaluating OAD. To solve this situation, Baptista et al. [37] redefined the OAD problem and introduced the novel Instantaneous Accuracy metric.

Online Action Detection is clearly not the scope of our work. However, as the authors stated in [37], online approaches should meet certain conditions that we proceed to incorporate to our online unsupervised AP method. For instance, our online proposals are causal, i.e., future information cannot be used for their predictions. Moreover, the proposals must be detected as soon as they happen, hence being dynamic: they are allowed to grow over time, as the video does.

## 3. Proposed Method

In this section, we first proceed to formally introduce the unsupervised AP approach, defining some needed mathematical notation (Section 3.1). We then, in Section 3.2, describe the problem of learning a SVC for the unsupervised setting. Finally, Section 3.3 introduces the novel filtering mechanism to refine the proposals.

### 3.1. Problem Setup

We assume that an untrimmed video can be denoted as a frame sequence $V^{i}={\left\{v_{n}^{i}\right\}}_{n=1}^{l_{i}}$, where $l_{i}$ encodes the duration of the video, and $v_{n}^{i}$ is the *n*-th RGB frame. This kind of videos contain portions without actions. Therefore, we define the temporal action annotations for $V^{i}$ as $A_{V^{i}}={\left\{a_{k}=\left(t_{1,k},t_{2,k}\right)\right\}}_{k=1}^{K_{V^{i}}}$, being $t_{1,k}$ and $t_{2,k}$ the starting and ending times for the action instance *k*, respectively, and $K_{V^{i}}$ the total number of actions instances in the video $V^{i}$.

The objective of an AP method is to generate a set of proposals $AP_{V^{i}}={\left\{ap_{k}\right\}}_{k=1}^{Kp}$ that correctly overlaps with the set $A_{V^{i}}$. While traditional supervised methods are allowed to use the action annotations to learn the AP models, an unsupervised approach cannot. So, the objective is to generate the set $AP_{V^{i}}$ without using any temporal annotation.

What is the hypothesis upon which our work is based? It is clear: frames that belong to different segments in a particular video could be separable by classifiers, once they have been projected to a set of features extracted with a deep network. Technically, we propose to use the ability to separate two sets of features that an SVC [38] exhibits to generate candidate segments for action proposals.

After generating candidate segments, we propose to consolidate them with a filter based on rank-pooling [39] and segment dynamics. If we consider that the actions have a distinguishable structure in their features, this module will try to find and discard those segments that do not present it and, therefore, would belong to the background.

**Figure 3:**
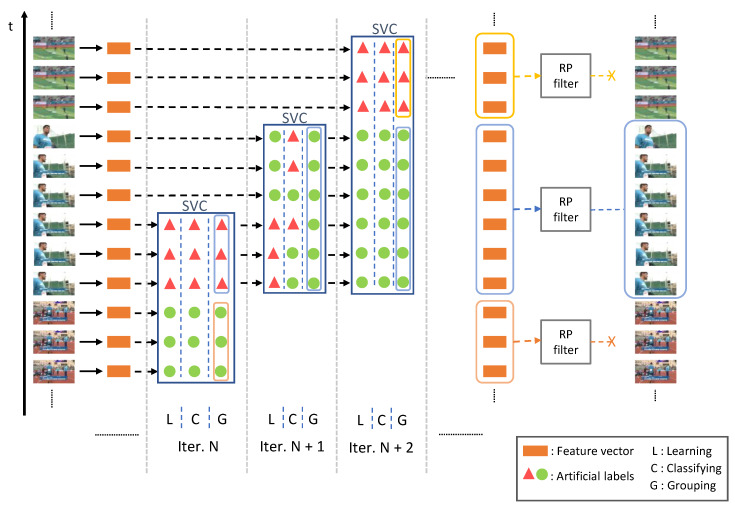
Unsupervised action proposals generation process. At each iteration, the SVC module is, first, fed with a certain number of contiguous frame features. Afterwards, they are artificially labeled for the learning step of the SVC module (note that the labels are artificial, independent at each step and do not correspond to any actual category). Finally, the SVC will decide on which groups to make after evaluating the classification error. A high value means that the features are not easily separable and, thus, they belong to the same segment. Conversely, features are considered from different groups when the SVC is capable of separating them. The rank pooling module will determine whether the candidate segments generated by the SVC are actual proposals. The model operates completely online, accessing only to the video frames available until time *t*.

This would be done in an unsupervised scenario where the deep network is pre-trained to recognize objects or actions in datasets that are different from the used one, and without any annotation, just feeding the approach with sets of contiguous video features. Figure 3 showcases the whole approach. The whole process is performed in an online way, where only information until time *t* is used.

### 3.2. Learning Unsupervised SVC for AP

Given the frame sequence $V^{i}={\left\{v_{n}^{i}\right\}}_{n=1}^{l_{i}}$, our model computes, first, any state-of-the-art feature representation for every frame or every set of frames, as our approach can actually generalize to these two types of representations. So, the sequence $V^{i}$ is converted to a set of visual features $F^{i}={\left\{f_{n}^{i}\right\}}_{n=1}^{l_{i}}$, where $f_{n}^{i}\in R^{d}$.

As depicted in Figure 3, a particular video is analyzed in an online fashion to find different segments on it. To do so, our approach is based on an online classification-based procedure using SVC [38], so we name our model as SVC-UAP, which stands for SVC Unsupervised AP. In a nutshell, the input features are, first, organized in two consecutive but different groups. Then, the SVC is trained to separate them. When this module confuses them, the features are considered similar and hence from the same group. Conversely, features are considered from different groups when the SVC is capable of separating them. The process iterates throughout the video to generate all candidate segments for action proposals.

Technically, our model begins to process the video $V^{i}$ by accessing the first 2×N features in $F^{i}$ to split them into into two sets of *N* consecutive features, $S_{t=1}^{+}$ and $S_{t=1}^{-}$, i.e., $S_{t=1}^{+}=\left\{f_{1}^{i},f_{2}^{i},\ldots ,f_{n}^{i}\right\}$ and $S_{t=1}^{-}=\left\{f_{n+1}^{i},f_{n+2}^{i},\ldots ,f_{2n}^{i}\right\}$. Note that t=1 because it is in the first iteration of the process and that for every new iteration *N* new features are evaluated. The results in Section 4.2.1 show the great importance of this parameter to our unsupervised model.

The following step consists in finding whether these two sets belong to the same segment. To do so, the two sets are artificially identified with two different labels $Y_{t=1}^{+}={\left\{+1\right\}}_{n=1}^{N}$ and $Y_{t=1}^{-}={\left\{-1\right\}}_{n=1}^{N}$, and the SVC proceeds to learn to separate them according to the labels. Mathematically, we define the tuple $\left(w_{t},b_{t}\right)$ which represents the max-margin hyperplane to separate $S_{t}^{+}$ and $S_{t}^{-}$. Our SVC solves the following primal problem to find $\left(w_{t},b_{t}\right)$:

(1) $$\min_{w_{t},b_{t},\zeta }\frac{1}{2}w_{t}^{T}w_{t}+C\sum_{k}\zeta_{k}\;,$$

(2) $$\begin{array}{cc} \mathrm{subject}\mathrm{to} & y_{k}\left(w_{t}^{T}\phi \left(f_{k}\right)+b_{t}\right)\geq 1-\zeta_{k},\\ \; & \zeta_{k}\geq 0\;\forall k\;, \end{array}$$

where $f_{k}\in R^{d}$ are the given *K* feature vectors in the sets $S_{t}^{+}$ and $S_{t}^{-}$, organized in two classes, with the labels of $Y_{t}^{+}$ and $Y_{t}^{-}$ concatenated in vector *y*. In Equation (2), we use the function ϕ() to implicitly map the training feature vectors to a higher dimensional space. Note that if a linear kernel is used, then $\phi \left(f_{k}\right)=f_{k}$. If a different kernel function $K(f_{k},k_{l})$ is used, then $K\left(f_{k},k_{l}\right)=\phi {\left(f_{k}\right)}^{T}\phi \left(f_{l}\right)$. Overall, we do not impose any constraint on the kernel to be used.

Once the SVC for iteration *t* has been learned and $\left(w_{t},b_{t}\right)$ obtained, the algorithm evaluates its actual performance on the provided features by measuring the classification error rate ($Cer_{t}$) for iteration *t*. Lastly, it decides whether to join or separate the initial groups of features. A high $Cer_{t}$ means that the SVC is not able to correctly separate the two sets. Hence, the two sets of features $S_{t}^{+}$ and $S_{t}^{-}$ should be joined in the same candidate proposal for the next iteration of the algorithm. On the other hand, a low $Cer_{t}$ implies that the set $S_{t}^{+}$ is different from $S_{t}^{-}$ and can be considered a different proposal. Therefore, we define the threshold α to evaluate these conditions: if $Cer_{t}\geq \alpha$ then $S_{t+1}^{+}=S_{t}^{+}⋃S_{t}^{-}$, the proposal size is increased for the next iteration; if $Cer_{t}<\alpha$, then $S_{t+1}^{+}=S_{t}^{-}$ and a new action proposal $ap_{k}$ is generated from the set $S_{t}^{+}$.

Note that standard evaluation metrics for AP require the models to assign a score for every generated proposal. We propose to use the $Cer_{t}$ for which the action proposal is generated: $s_{k}=e^{-Cer_{t}}$. With the solution described so far, we can evaluate the recall over the annotations just by ordering the generated candidate proposals and selecting the set of *n* best proposals, according to this score.

The whole process is shown in Figure 3. It can be seen how in each iteration the SVC module decides on which groups to make after learning and classifying based on the initial artificial labels.

### 3.3. Rank-Pooling Filtering

Through the SVC-UAP, every video feature is assigned to a particular candidate action proposal, although with a different score. We need an unsupervised mechanism to further filter those segments that belong to the background. To this aim, we propose using the dynamics of the segments to consider them as action or background.

The dynamics of a certain video can be defined as the video-wide temporal evolution of the appearance of its frames. This type of meta-features has been used to address the problem of action recognition in several works [39,40,41,42,43,44,45]. Here, instead, we propose using this concept to find and discard the background segments in an unsupervised way. We work with the hypothesis that actions may have a temporal structure, while the background does not. If the dynamics of a segment are compared to those of the same segment but when its features have been randomly disordered, their similarity will be high only for background. Conversely, if the same is done with an action segment, the effect on the dynamics will be much more visible as the randomized version of the segment will miss the structure of the action.

Let $ap_{k}$ be a candidate action proposal generated by the SVC module. First, we build the set $F^{ap_{k}}={\left\{f_{n}\right\}}_{n=1}^{l_{ap_{k}}}$, where $f_{n}\in R^{d}$ and $l_{ap_{k}}$ encodes the size of the proposal. $F^{ap_{k}}$ contains the ordered set of features for the video frames included in $ap_{k}$. Then, we create the set of features ${\tilde{F}}^{ap_{k}}$, which is a randomly disordered version of $F^{ap_{k}}$. Finally, we leverage the rank-pooling model of [39] to compute the dynamics of $F^{ap_{k}}$ and ${\tilde{F}}^{ap_{k}}$.

As in the rank-pooling model, the temporal dynamics of a set of features is summarized as the parameters of a curve in the input space that captures the frame temporal order via linear projections. This is done by optimizing a pairwise-learning-to-rank problem based on SVM. In particular, we implement a rank-SVM with a linear Support Vector Regression (SVR) based formulation, which is known to be a robust point-wise ranking method [46].

Given any set of features $F={\left\{f_{t}\right\}}_{t=1}^{l}=\left\{f_{1},f_{2},\ldots ,f_{l}\right\}$, we search for a direct projection of the input vectors $f_{t}$ to a time variable *t* employing a linear model with parameters $\omega^{F}$, as follows:

(3) $$\omega^{F}=\underset{\omega^{F}}{arg min}\sum_{t}\left|t-\omega^{F}\cdot f_{t}\right|\;.$$

This way, $\omega^{F}$ summarizes dynamics sequence, becoming the pooled dynamics descriptor for F, which compactly encodes a sequence of features into a single vector.

For the implementation, the following operations are applied to the feature vectors in F before computing the rank-pooling dynamics $\omega^{F}$: (1) a temporal smoothing with time varying mean vectors; (2) a point-wise non-linear operator Φ(·); and (3) an L2 normalization. For the experiments, we have chosen the following non linear function: $\Phi \left(f_{t}\right)=\mathrm{sgn}\left(f_{t}\right)\sqrt{|f_{t}|}$.

The rank-pooling filtering mechanism computes the dynamics for $F^{ap_{k}}$ and ${\tilde{F}}^{ap_{k}}$, being them $\omega^{F^{ap_{k}}}$ and $\omega^{{\tilde{F}}^{ap_{k}}}$, respectively. As we described above, the distance between these two dynamic vectors allows our model to identify action proposals, discarding candidates that might include background information. For a candidate that does not contain any action inside, the distance between its dynamics and the dynamics of its randomized version should not be high. We implement this filtering mechanism by measuring the Euclidean distance between these two vectors and using a threshold *r* to discard background segments: if $d(\omega^{F^{ap_{k}}},\omega^{{\tilde{F}}^{ap_{k}}})<r$, the candidate proposal is rejected.

As a summary, we want to emphasize that the whole filtering process based on these dynamics, obtained through rank-pooling, works in a fully unsupervised way. The dynamics computation does not require access to any type of annotation. Additionally, the implementation based on linear SVR is efficient, which allows our model to work on online video streams.

Apart from the visual description of Figure 2, we also describe our proposed method in Algorithm 1. Specifically, it shows the procedure that our SVC-UAP solution follows to obtain the action proposals of a certain video.

$$F^{i}={\left\{f_{n}^{i}\right\}}_{n=1}^{l_{i}}$$

**Algorithm 1** SVC-UAP method on a certain video to obtain its action proposalsGiven a certain video $V^{i}$**Input:** Incoming frames of a certain video: $V^{i}=\left\{v_{0}^{i},v_{1}^{i},v_{2}^{i},\ldots ,v_{l_{i}}^{i}\right\}$        Features to collect in each iteration: *N*        Cer threshold: α        Rank Pooling filter threshold: *r***Output:** Set of action proposals: $AP_{V^{i}}={\left\{ap_{k}\right\}}_{k=1}^{Kp}$1: AP={}2: f=03: **while** not end of video **do**4:     **if** first iteration **then**5:         $frames=\{v_{f}^{i},v_{f+1}^{i},v_{f+2}^{i},\ldots ,v_{f+2*N}^{i}\}$▹ First incoming video frames6:         features=FeatExtr(frames)▹ Feature extraction7:         $S_{1}=features\left[0:N\right]$
▹ Split in two set of features8:         $S_{2}=features\left[N:2*N\right]$9:         f=2*N+110:     **else**11:         $frames=\{v_{f},v_{f+1},v_{f+2},\ldots ,v_{f+N}\}$▹ Next incoming video frames12:         features=FeatExtr(frames)13:         $S_{2}=features$14:         $S_{1}=S_{previuous}$15:         f=f+N+116:     **end if**17:     $Cerr=SVC(S_{1},S_{2})$▹ Train and apply SVC module and get classification error rate18:     **if**
Cerr≤α **then**▹ No action proposal found19:         $S_{previous}=Join\left(S_{previous},S_{1}\right)$▹ Join the two sets20:     **else**▹ Possible action proposal found21:         $S_{randomized}=randomize\left(S_{previous}\right)$▹ Randomly shuffles the input set22:         $distance=L2(RP\left(S_{previous}\right),RP\left(S_{randomized}\right))$▹ Distance between rank pooling vectors23:         **if**
distance≤r
**then**24:            $discard(S_{previous})$▹ If similar, it is background. Thus, candidate discarded25:         **else**26:            $AP=append(AP,S_{previous})$▹ Proposal confirmed27:            $S_{previous}=\left\{\right\}$28:         **end if**29:     **end if**30: **end while**31: **return** AP

## 4. Experiments

### 4.1. Experimental Setup

#### 4.1.1. Datasets

We evaluate for the first time an unsupervised solution on the two main datasets for the AP problem: ActivityNet [26] and Thumos’14 [27].

**ActivityNet.** It is, in its 1.3 version, a large-scale dataset that provides more than 19k annotated untrimmed videos (648 h of videos). This means that not all the video frames belong to actions, but also to background. Note that this is not the case in other recent large-scale video dataset, e.g., Kinetics-600 [47] or Moments in Time [48]. Additionally, this dataset has been used as a benchmark for comparison of state-of-the-art supervised AP models. This fact offers us the opportunity to compare how far our unsupervised solution is from the state of the art.

**Thumos’14.** This dataset comprises more than 400 untrimmed videos, where 20 action categories have been annotated. We follow the common setup for AP: we train and test on the validation and test subsets, respectively. Compared to ActivityNet, Thumos’14 is not a large-scale dataset. However, it is also a benchmark for several AP models, e.g., [15,16,17]. Therefore, we have decided to integrate it into our experimental setup to validate the performance of our unsupervised AP model, establishing a novel reference for the unsupervised paradigm of the AP task. Furthermore, using Thumos’14 allows us to have a direct comparison with state-of-the-art models that provide results for the supervised setting.

#### 4.1.2. Evaluation Metric

During the evaluation, we follow the standard and use the Average-Recall versus Average Number of Proposals per Video (AR-AN) metric of the ActivityNet challenges [11]. The objective of any AP method is to produce temporal segments where an action might be occurring. Given a set of proposals, this metric considers a true positive ($t_{p}$) if the proposal segment has a temporal intersection over union tIoU with the annotated action that is greater than a certain threshold. The recall is computed following the next equation:

(4) $$R=\frac{t_{p}}{t_{p}+f_{n}}\;,$$

where $f_{n}$ stands for false negative.

As in the official ActivityNet challenge for AP, we define the Average-Recall (AR) as the mean of the recall values computed for the set of tIoU thresholds [0.5,0.95], using a step of 0.05. Note that for Thumos’14, tIoU thresholds are [0.5,1.0], using a step of 0.1. On the other hand, we define the Average Number of Proposals per video (AN) as the total number of action proposals divided by the number of videos. We use 100 values for AN, from 0 to 100, when plotting the AR-AN curve. For the comparison of methods, we report the area under the AR-AN curve (AUC), as it is done in the ActivityNet Challenge. The same metric is also used with the Thumos’14 dataset as it has recently been done in other works [15,16,17].

#### 4.1.3. Implementation Details

We have implemented the whole approach on Python, using the excellent scikit-learn library [49]. We publicly release all the code and data needed to reproduce the reported results (Code is available on our public repository at https://github.com/gramuah/svc-uap).

As a fully unsupervised approach, we do not use any annotation provided with the datasets. We directly run our solutions on the 4926 and 210 videos provided in the validation and test subsets of ActivityNet and Thumos’14, respectively. For the evaluation, we do not exclude any of the action categories annotated in ActivityNet and Thumos’14.

As it has been described in Section 3, our approach is agnostic to the backbone kernel used in the SVC. Specifically, during the experiments we use linear and RBF models. The influence of their respective parameters is analyzed in an ablation study using ActivityNet dataset. The rank-pooling based filtering is implemented by applying the temporal smoothing to the input data, as well as the non-linearity function detailed in Section 3.3. Moreover, before learning the linear regressor to obtain the corresponding dynamics, the data are l2 normalized. The base name of our approach is SVC-UAP. In Table 1 we show the variants of our approach that have been used in the experiments. We name them depending on the components that are used. If the linear kernel is used, we employ the acronym SVC-UAP-linear. If the rank-pooling is integrated in, we refer to our solution as SVC-UAP-linear-rp. When the RBF kernel is used, the correspondent acronyms are: SVC-UAP-RBF and SVC-UAP-RBF-rp.

As a sanity check baseline, we also implement the method RAND, which generates random action proposals. It technically generates a set of random time intervals as proposals within a video and assigns random scores to them.

As for features, all approaches use the same pre-computed input features. They consist of the activations of the second fully-connected layer (named fc7) of the publicly available C3D network [28]. Specifically, we use this model with a temporal resolution of 16 frames and eight overlapping frames, hence we extract features for every eight frames. For ActivityNet, we follow what was proposed in the ActivityNet challenge and reduce with PCA the dimensionality of the fc7 layer from 4096 to 500. For Thumos’14 we do not apply any reduction to the vector size. It is very important to note that the features used are fine-tuned on the Sports1M dataset [50] and not on any of the used datasets. This way, we avoid any type of model/feature selection for specific data. Otherwise, it could be considered a violation of the unsupervised character of our work.

### 4.2. Experimental Evaluation in Activitynet

#### 4.2.1. Ablation Study

In this section, we analyze the influence of each of the parameters and parts of our SVC-UAP model. All the experiments that have been conducted, and the involved parameters, are described in Table 2.

**Figure 4:**
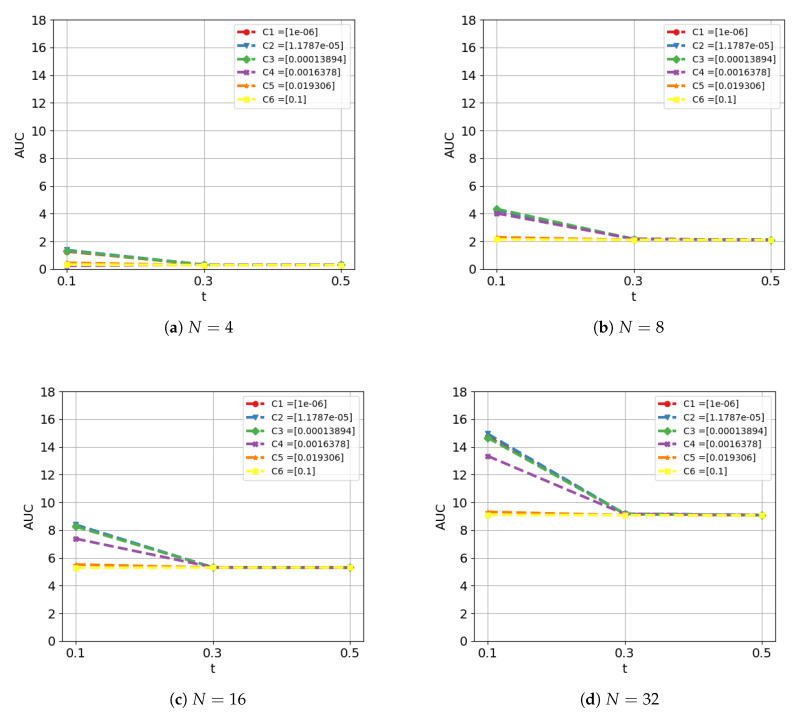
Ablation study on ActivityNet dataset. Performance in terms of AUC of the AR-AN curve when varying the parameters *N*, *t* and *C*. Basic configuration: linear kernel for the SVC module and no rank-pooling filter.

**Experiment 1. Influence of main parameters.** We first study the influence of the main parameters of the model beginning from a basic configuration with a linear kernel for the SVC module and no rank-pooling filter. The evaluated parameters are: the number of samples added in each iteration (*N*), the threshold for the online clustering/aggregation (*t*) and the regularization parameter of the linear kernel (*C*) of the SVC. The range of values used for each parameter is shown in the Experiment 1 description in Table 2. Figure 4 shows the evolution of the AUC when varying the parameters.

From Figure 4, we can draw the following conclusions. First, the influence of the threshold *t* remains stable for all *N* and *C*: the higher this threshold is, the more restrictive our model is when generating action proposals. We can conclude t=0.1 offers optimal results. Second, the analysis reveals the best performance is obtained when $C=1.1787\times 10^{-5}$. Interestingly, this holds for all the evaluated combinations of parameters. Finally, the parameter *N* makes the difference. The performance increases for higher values of *N*, i.e., incorporating bigger groups of features at each iteration appears more beneficial. We shall discuss more this phenomenon below, after including the rank-pooling filter. We continue the ablation study using the winner configuration of parameters from the previous analysis, that is: $C=1.1787\times 10^{-5}$, N=32 and t=0.1.

**Figure 5:**
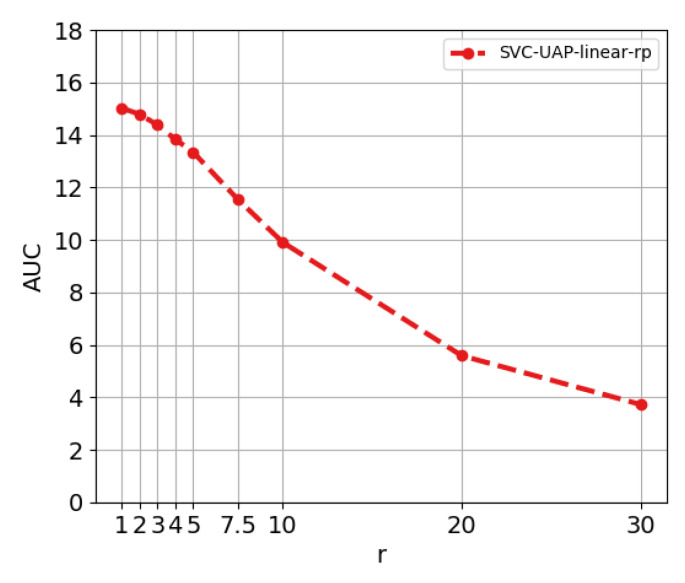
Evolution of the AUC of the AR-AN curve when varying the threshold *r* of the rank-pooling filter. The model is configured to use a linear kernel for the SVC module.

**Experiment 2. Adding the rank-pooling filter module.** The next objective is to validate the effectiveness of the rank-pooling based filter module. Figure 5 showcases the variation in the AUC of the AR-AN curve when the rank-pooling is integrated. We explicitly validate the influence of the rank-pooling parameter *r*. High values of this threshold lead to a more restrictive rank-pooling filter which discards more candidate proposals. Conversely, lower values mean that fewer proposals are discarded. As we are in an unsupervised scenario, we have to trust the features and assume that action and background are differentiable enough, though there may exist action features similar to that of background. As we cannot control this, we have to find a balance situation. Such situation appears when r∈[1,4]: the rank-pooling filter is able to discard proposals without dramatically losing performance. Table 3 shows the difference in the performance when adding this module. Although there is a slight improvement in AUC, the filter also improves our approach from the perspective of efficiency. The fact of having the same or better performance with fewer proposals suggests that the precision of the method is increasing.

**Figure 6:**
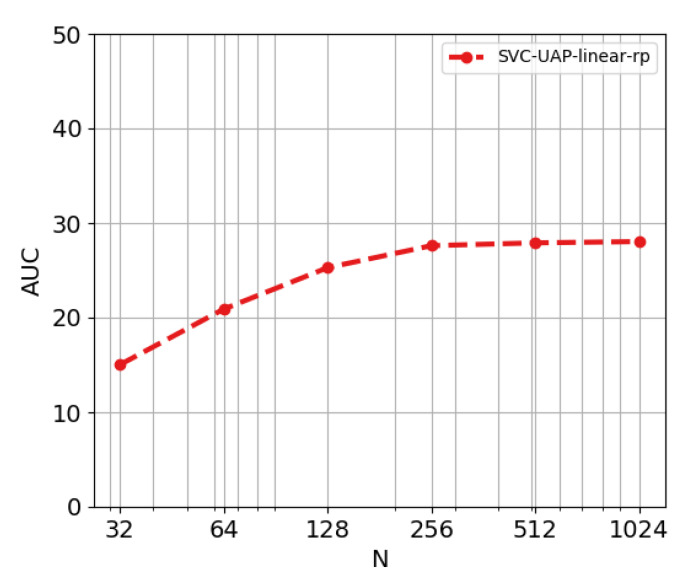
Evolution of AUC of AR-AN curve when *N* increases.

**Experiment 3. Influence of parameter N.** As we pointed above, in light of the results in Figure 4 and Figure 6 configuring the parameter *N* seems very relevant to have a good performance. Specifically, Figure 6 shows the performance of the SVC-linear-rp model for N=[32,64,128,256,512,1024], where the AUC reaches a plateau for N≥256. Analyzing the statistics of the ActivityNet dataset, one discovers that: (a) each video has on average 3 annotated actions; and (b) annotations cover 55% of the duration of the videos. These two facts mean that actions in this dataset are long. Therefore, generating longer proposals results more beneficial. This parameter controls how big the new group of features that is incorporated in each iteration is and, thus, it is indirectly controlling the duration of the proposals.

Overall, the use of each of the modules that have been proposed in this work is supported by the results reported in this ablation study. For the rest of the experiments on this dataset, we use the discovered optimal parameters.

#### 4.2.2. Main Results

**Figure 7:**
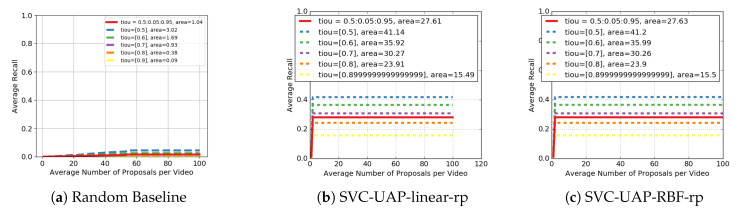
Performance in terms of AR-AN for the problem of Action Proposals in AcivityNet dataset. Comparison of the proposed approach with different kernels for the SVC module, including the random baseline.

This section presents the main results for the problem of unsupervised AP. Figure 7 includes the performance of our approach when using different kernels for the SVC module, as well as that of the RAND baseline. This last random model achieves only an AR of 1.04, which means that AP on ActivityNet is a complex task. From Figure 7b,c we conclude that for this dataset the RBF and linear kernels offer the same performance. For this reason, and based on efficiency, we conclude that it is more practical to use the approach with the linear kernel, especially for an online setting.

It is worth recalling that the proposed approach works in an online fashion, which means that the generated proposals do not overlap, their number being lower than the number that the offline proposals usually provide. This leads to always having a plateau after a few AN, as seen in Figure 7b,c.

#### 4.2.3. Comparison to State-of-the-Art Supervised AP Models

Table 4 shows the performance in terms of AUC in the AR-AN curve for the current AP state-of-the-art supervised models. It is observed that the best supervised model achieves 67.10 of AUC@100 proposals. Thus, our fully unsupervised models are able to recover 41% of the performance of this model, showing a promising direction for unsupervised approaches.

Note that all state-of-the-art models present an offline setting where the full video must be analyzed to cast proposals. Additionally, these methods generate thousands of overlapped AP per video. On the contrary, our solutions work completely online, generating proposals as videos evolve and in a more efficient way since the number of AP is, by far, smaller.

### 4.3. Experimental Evaluation on Thumos’14

**Figure 8:**
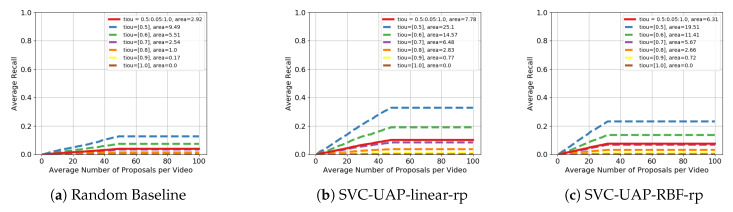
Performance in terms of AR-AN for the problem of Action Proposals in Thumos’14 dataset. Comparison ofthe proposed approach with different kernels for the SVC module, including the random baseline.

For the sake of a thorough experimental evaluation, we also report the performance of our unsupervised models on the Thumos’14 dataset. We experiment with linear and RBF kernels for the SVC module and the values for the parameters are: N=8, t=0.09, C=0.019306 and r=0.1. As with the previous dataset, we include in Figure 8 the AR-AN curves of our approach when using different kernels, as well as that of the random baseline. Additionally and following what is done in the literature, we present in Table 5 the results in terms of AR-AN with a maximum of 50 and 100 proposals per video (AR@50 and AR@100).

Figure 8 shows that the gap in the performance between our solutions and the baseline is smaller than that reported in ActivityNet. This means that: (i) the random model is adequate as a baseline for the problem and (ii) this dataset is even more challenging than ActivityNet.

**Figure 9:**
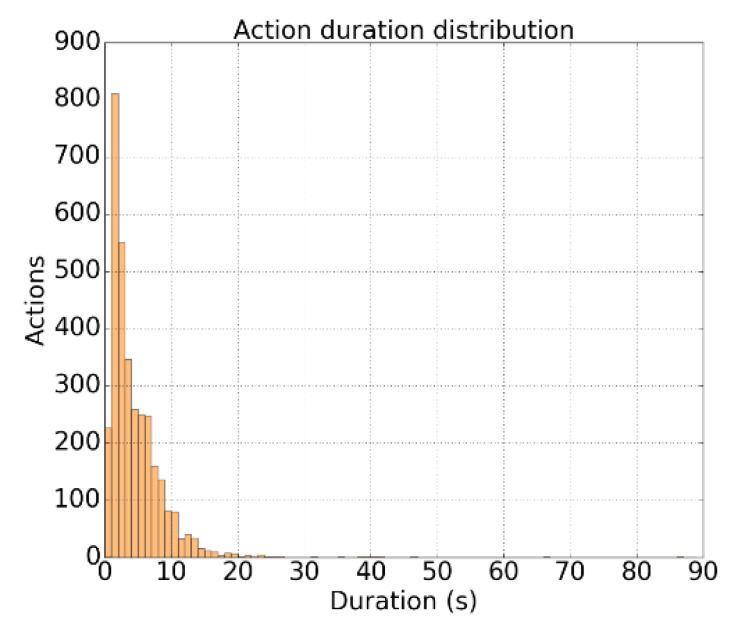
Ditribution of the duration of the actions in Thumos’14.

We have also analyzed some statistics of the dataset to better understand the results. First, the average duration of the annotated actions is less than 5 s, as it is shown in Figure 9. Second, there are about 15 instances per video, covering only 20% of the content. These numbers indicate that Thumos’14 is a much more sparse dataset with shorter action segments than ActivityNet. The sparsity of the dataset is consistent with the shape of the curves in Figure 8b,c. This fact suggests that AP methods need to throw more proposals to improve the recall, but the online condition of our approach clearly increases the challenge. While it cannot apply any post-processing to the generated proposals, many offline state-of-the-art methods do it to maximize the recall. Remember that in an offline setting, the approaches have access to the whole video. We do not find this maximization absolutely necessary, as precision can be forgotten, which in an online scenario would be disastrous.

As for the comparison to the state of the art, our solution with a linear kernel achieves a 10.16 of recall value with only 50 proposals per video and without any supervision. This represents 59% and 26% of the performance of the worse (SCNN-prop [12]) and the better (BMN [17]) supervised state-of-the-art models, respectively. These results are sufficiently motivating to continue investigating on the unsupervised paradigm for action proposals.

## 5. Conclusions

Throughout this work we have presented a simple, unsupervised, online and efficient classification-based method for the problem of APs. This approach generates candidate action proposals through an SVC, capable of grouping consecutive set of frame features in a certain video to create time boundaries that define action candidate segments. We have also introduced a filtering module which uses the rank-pooling over the dynamics of the candidates segments to discard those that belong to the background of the video. It is important to note that we do not apply any level of supervision to the model: no action annotations are used, as well as the feature extraction network has not been pre-trained in any of the used datasets.

To the best of our knowledge, it is the first time that a thorough experimental evaluation of an unsupervised approach is presented on the two main benchmarks for Action Proposals: ActivityNet and Thumos’14. The ablation study that has been conducted on the ActivityNet dataset justifies the integration of each part of our approach and supports our working hypothesis. Although the datasets show different natures in their annotations, our solution is capable of adapting to both of them. When comparing to the state of the art, our best configuration achieves more than 41% and 26% of the recall performance of the best supervised models for ActivityNet and Thumos’14 datasets, respectively.

Video datasets are growing enormously and, consequently, their labeling becomes a very expensive task. Having systems that can work without requiring labels of any kind, as the one we have proposed here, is of great interest. This fact coupled with the online nature of our method, makes us conclude that we have proposed a promising new paradigm for AP. Different from the current state-of-the-art approaches, ours could provide the proposals as they are obtained to a certain action classifier and detect the action as soon as it occurs. In fact, as a future work, we plan to explore the impact of our unsupervised action proposals on the Temporal Action Detection task.

We will make our code and results publicly available so that others can reproduce our results and explore this novel unsupervised AP perspective.

## Figures and Tables

**Table 1:** Variants of our SVC-UAP method used in the experiments.

	Linear Kernel	RBF Kernel	Rank Pooling
SVC-UAP-linear	✓	✗	✗
SVC-UAP-linear-rp	✓	✗	✓
SVC-UAP-RBB	✗	✓	✗
SVC-UAP-RBF-rp	✗	✓	✓

**Table 2:** This table contains a brief description of each of the experiments that have been conducted in the ablation study.

	Modified Parameters	Fixed Parameters	SVC-UAP Variant
	t=[0.1,0.3,0.5]		
**Experiment 1**	N=[4,8,16,32]	-	SVC-UAP-linear
	$C=[1\times 10^{-6},1.1787\times 10^{-5},1.3894\times 10^{-4},1.6378\times 10^{-3},1.9306\times 10^{-2},1\times 10^{-1}]$		
		t=0.1	
**Experiment 2**	r=[1,2,3,4,5,7.5,10,20,30]	$C=1.1787\times 10^{-5}$	SVC-UAP-linear-rp
		N=32	
		t=0.1	
**Experiment 3**	N=[32,64,128,256,512,1024]	$C=1.1787\times 10^{-5}$	SVC-UAP-linear-rp
		r=1	

**Table 3:** Comparison between SVC-UAP-linear (without rank-pooling) and SVC-UAP-linear-rp (with rank-pooling).

	SVC-UAP-Linear	SVC-UAP-Linear-rp
AUC	14.94	**15.02**

**Table 4:** Comparison with the state-of-the-art for the problem of AP in ActivityNet. ^s^ indicates the method is supervised.

	AUC
[51]^s^	59.58
[52]^s^	64.40
CTAP [15]^s^	65.72
BSN [16]^s^	66.17
BMN [17]^s^	67.10
SVC-UAP-linear-rp	27.61
SVC-UAP-RBF-rp	27.63

**Table 5:** Comparison with the state-of-the-art for the problem of AP in Thumos’14. ^s^ indicates the method is supervised.

	AR@50	AR@100
SCNN-prop [12]^s^	17.22	26.17
SST [21]^s^	19.90	28.36
CTAP [15]^s^	32.49	42.61
BSN [16]^s^	37.46	46.06
BMN [17]^s^	39.36	47.72
SVC-UAP-linear-rp	10.16	10.16
SVC-UAP-RBF-rp	7.53	7.53
Random baseline	3.96	3.96

## Abbreviations

The following abbreviations are used in this manuscript:

TAD	Temporal Action Detection
AP	Action Proposal
OAD	Online Action Detection
SVM	Support Vector Machine
SVC	Support Vector Classifier
RBF	Radial basis function
SVC-UAP	Support Vector Classifiers for Unsupervised Action Proposals
SVR	Support Vector Regression
PCA	Principal Component Analysis
